# Plasma levels of zinc, selenium, and copper among incarcerated men in Switzerland

**DOI:** 10.3389/ijph.2026.1609553

**Published:** 2026-08-04

**Authors:** Marie Bettex, Audrey Dettwiler, Bastien Trachsel, Constantin Bondolfi, Julia Spaltenstein, Mette M. Berger, Aurélien Thomas, Sébastien Lenglet, Serge Rezzi, Nadia Fucina, Didier Delessert, Patrick Bodenmann, Murielle Bochud

**Affiliations:** 1 Unisanté, University Center for Primary Care and Public Health, Lausanne, Switzerland & University of Lausanne, Lausanne, Switzerland; 2 Universite de Lausanne Faculte de Biologie et de Medecine, Lausanne, Switzerland; 3 Unit of Forensic Toxicology and Chemistry, CURML, Lausanne and Geneva University Hospitals, Lausanne, Geneva, Switzerland; 4 Swiss Nutrition and Health Foundation, Lausanne, Switzerland; 5 Service of Prison Medicine and Psychiatry, Centre Hospitalier Universitaire Vaudois, Lausanne, Switzerland

**Keywords:** male, micronutrient status, nutrition, plasma, prisons, selenium deficiency

## Abstract

**Objectives:**

Incarcerated people carry many health problems. While prisons provide structured healthcare and meals, micronutrient status remains unclear. We assessed plasma zinc, selenium, and copper status in incarcerated men in Vaud (French-speaking canton in Switzerland) and explored prevention-relevant patterns.

**Methods:**

Cross-sectional study (May-September 2023) of incarcerated men in three prisons. Participants (n = 206) completed a health-related questionnaire and provided venous blood. Plasma trace elements were measured by inductively coupled-plasma-mass spectrometry. Associations with individual characteristics were examined and differences between-prisons were assessed. Micronutrient concentrations were compared with community-men, adjusting for age and body mass index.

**Results:**

Prevalence of men with micronutrient plasma level below the normal range did not differ between incarcerated and community men. Adjusted means showed modestly lower selenium and higher zinc and copper in men experiencing incarceration. Within prisons, lower selenium was associated with older age, lower pre-incarceration income, and smoking. Zinc was lower with age and in one facility.

**Conclusion:**

Overall micronutrient status was reassuring, yet the heterogeneity, possibly reflecting individual vulnerabilities and institutional conditions, supports continued, facility-aware monitoring to identify at-risk groups and protect health.

## Introduction

Incarcerated individuals represent a marginalized and vulnerable population, yet incarceration can also represent an opportunity to reduce health inequalities by providing consistent medical support to a population that often lacks access to care in the community [1–9], as well as opportunities to promote health through preventive measures such as more balanced nutrition and access to physical activity [10, 11].

Although institutional meals are typically provided in prisons, the actual nutritional intake can vary widely. Many incarcerated people rely on supplementary foods from canteens or external sources, often choosing items that are energy-dense but nutritionally poor [5, 12, 13]. This pattern can contribute to both weight gain and nutritional imbalances, increasing the risk of chronic diseases like obesity, diabetes, and cardiovascular disease. Weight gain is further compounded by the frequent use of psychotropic medications, particularly antipsychotics, which are known to induce metabolic changes and increase appetite [14]. Additionally, the social and psychological dynamics of prison life can significantly shape eating habits, with food serving as a source of comfort and a form of resistance to institutional control [15].

Given these factors, it remains unclear to what extent the dietary patterns observed in prisons influence the nutritional status of incarcerated persons, particularly regarding essential trace elements like zinc, selenium, and copper. These micronutrients are critical for immune function, oxidative stress control, and metabolic regulation, making their assessment particularly relevant for this population [16–19]. While national programs, coordinated by the Federal Food Safety and Veterinary Office, monitor these trace elements in the general population [20–22], comparable data for people in detention are lacking, underscoring the importance for such evaluation.

Canton de Vaud has six custodial facilities: four adult prisons (three for men, one for women, covering remand and sentence execution), one for minors/young adults, and one semi-detention establishment: three sites were included in the present study. In November 2023, Vaud accounted for 10.7% of Switzerland’s total detention population, with 110 per 100 000 inhabitants, above the national average (∼76/100 000) [23] and near the European median (∼105/100 000) [24]. Like most European countries, Switzerland, and Vaud in particular, has a predominantly male prison population, with men accounting for 94.1% of this population [25]. Vaud faces chronic overcrowding with 117% occupancy in 2023 versus 94% nationally [23]. This high-volume and high-occupancy context provides a pertinent setting for interpreting health and nutrition indicators in incarcerated population.

During the COVID-19 pandemic, Vaud cantonal health authorities launched the “SérocoVID” project to estimate prior SARS-CoV-2 infection, assess immunity, and identify transmission factors within its population. As part of this effort, three prison facilities located in the canton of Vaud (French-speaking Switzerland) were included due to their outbreak vulnerability, providing a unique opportunity to collect broader health data from incarcerated individuals, including plasma levels of zinc, selenium, and copper, beyond the study’s primary focus on viral transmission and immunity.

The primary aim of this study is to describe plasma zinc, selenium, and copper levels among incarcerated men in Canton of Vaud. We then compare these findings with data from men in the general Swiss adult population of the same region to identify potential differences and disparities. We also explore whether selected sociodemographic and behavioral factors are associated with plasma trace element levels among incarcerated individuals. Describing plasma trace element concentrations and their distribution in this population may help inform future monitoring and generate hypotheses for further research on nutritional and health determinants in prison settings.

## Methods

### Study design and setting

We conducted an observational cross-sectional study between May and September 2023 in three prisons in the Canton of Vaud, Switzerland, including incarcerated men housed in these facilities.

### Study population and recruitment

Participants were recruited through multilingual flyers distributed by the medical team during routine care or through prison mail, with additional information on posters and internal television broadcasts. Interested individuals returned a response slip and were invited to a study visit with research nurses to provide informed consent and completed data collection.

Exclusion criteria included inability to provide informed consent, unavailability, insufficient language proficiency in one of the translated study languages, or refusal to participate. A total of 206 individuals were enrolled (targeted n = 200), with only one withdrawal post-inclusion. The sample comprised 65 participants (31.6%) from Prison 1, 61 (29.6%) from Prison 2, and 78 (37.9%) from Prison 3. Facilities differed by incarceration regime: Prison 1 included pretrial and sentenced men; Prison 2 mainly individuals in pretrial detention, with a minority executing sentences; Prison 3 served exclusively for sentence execution. Daily routines and access to activities varied accordingly. Men in pretrial detention typically spend up to 23 h per day confined to their cells with limitation or no access to work. Additionally, Prison 2 faces chronic overcrowding, further affecting daily routines and living conditions.

Details regarding food provision systems and access to meals and canteen products were collected through discussions with prison staff and complemented by reports from the Swiss National Commission for the Prevention of Torture, which regularly monitors conditions in Swiss detention facilities [26]. These sources provided contextual information but did not constitute a formal nutritional assessment of institutional food provision or actual food intake.

Due to high turnover and logistical constraints, the number of individuals who received study information was undetermined. Healthcare staff redistributed study materials as needed, and some interested individuals were released before inclusion. The canton’s only women’s prison was under renovation and not included.

### Data collection and laboratory analyses

Participants completed a self-administered questionnaire capturing sociodemographic, health, and lifestyle factors, available in 9 languages (French, English, Spanish, German, Portuguese, Romanian, Albanian, Arabic, and Russian). Study nurses were available to assist as needed. Venous blood samples were collected on-site by trained nurses before 12AM (relation to food intake was not recorded) and transported the same day to Lausanne University Hospital (CHUV).

Trace elements quantification was done by the CHUV’s Forensic Medicine Center. using certified inductively coupled plasma mass spectrometry (ICP-MS) on an Agilent 7,700 instrument (Agilent Technologies, Santa Clara, USA) equipped with quadrupole detector and with integrated auto sampler. Detailed analytical parameters are available elsewhere [27, 28]. Isotopes measured were ^63^Cu, ^66^Zn, ^82^Se [2]. Samples were prepared by dilution 1/10 (vol/vol) with a HNO_3_ (1%), N-butanol (0.5%), Triton X-100 (0.1%) solution containing rhodium (Rh) and indium (In) (10ng/mL each) as internal standards. Batches were processed with a 6-points calibration curve and certified reference materials used as internal quality controls. Calibration solutions and internal standards were purchased from LabKings (Hilversum, Netherlands), nitric acid and Triton X-100 from Merck (Darmstadt, Germany) and N-butanol from VWR Chemical (Rosny-sous-Bois, France). Certified reference materials (plasma) were ClinChek® Controls, bought from RECIPE (München, Germany). Recoveries of trace elements from ClinCheck® Controls, the certified reference material, were 1.2% for repeatability (CVr) and 3.9% for reproducibility (CRV) for zinc at a level of concentration of 1160 mg/L, whereas the corresponding numbers were 1.4% and 2.5% at a concentration of 81.4 mg/L for selenium and 1.7% and 3.1% at a concentration of 692 for copper [27].

Primary analysis used male adult-specific reference intervals derived from the Swiss Kidney project on genes in hypertension (SKIPOGH, 2009–2012): Zn 620–999 ng/mL, Se 86–144 ng/mL, Cu 594–1141 ng/mL (women and <18 years excluded for comparability) [29]. These population-based intervals reflect distribution in Vaud, Geneva, and Bern not clinical outcomes or physiological requirements [27].

Given ongoing discussion in Switzerland about the adequacy of local cut-offs, we applied alternative thresholds, commonly used in clinical/research context, to report low-value prevalence: Zn < 700 ng/mL (recommended by the International Zinc Nutrition Consultative Group (IZiNCG) [30], the Biomarkers of Nutrition for Development (BOND) Expert Zinc Panel [31], the Scottish Trace Element and Micronutrient Diagnostic & Research Laboratory (STEMDR) [29, 32–35]), Cu < 636 ng/mL (also used by STEMDR [29, 33, 36]), Se < 60 ng/mL (ESPEN’s physiologically anchored cut-off linked to glutathione peroxidase activity plateau [37], also adopted by STEMDR and UK National Diet and Nutrition Survey [29, 33, 34]).

Ultra-sensitive C-reactive protein (CRP) was measured by the central CHUV laboratory using validated methods.

### Comparative population data

To assess differences with the general population, we used data from the NutriSerocovid study [38], a population-based survey from the Canton of Vaud comprising 751 randomly selected individuals identified via the Federal Office of Public Health and recruited between May and June 2020 (women and those under 18 were excluded).

### Representativeness assessment

To evaluate the representativeness of our sample, we compared key sociodemographic characteristics with national incarceration data published by the Swiss Federal Statistical Office (FSO) for 2023. Specifically, we used sanction execution data, stratified by nationality, sex, and residency status [39–41].

### Statistical analyses

Statistical analyses were performed using R (version 4.3.2). Descriptive statistics summarized participant characteristics and plasma concentrations of selenium, copper, and zinc. Continuous variables were described using percentiles (5th, 25th, 50th, 75th, 95th), as well as minimum and maximum values; categorical variables were summarized as frequencies and percentages. Missing data were documented, and no imputation was applied.

#### Comparison with men from the general population

Comparisons between incarcerated men and men from the general population were conducted using chi-squared tests for differences in deficiency prevalence, Student’s t-tests for unadjusted differences in means, and both the median test and Wilcoxon rank-sum test for unadjusted differences in medians. To account for potential confounding by age and body mass index (BMI), two known factors associated with micronutrient levels, we used multivariable linear regression models to estimate adjusted mean differences and quantile regression (at the 0.5 quantile) to assess adjusted differences in medians. P-values for adjusted mean differences were derived from t-tests on regression coefficients, while adjusted median differences were tested using Wald statistics in quantile regression models. Adjustment was limited to age and BMI due to the lack of additional sociodemographic variables in the general population sample.

#### Exploration of differences across prison facilities

Given structural differences between the three facilities, such as variation in detention regimes, meal provision systems (Prisons 1 and 3 share the same menu, Prison 2 operates its own kitchen), and general incarceration conditions, we assessed whether sociodemographic characteristics and trace element levels differed across prison sites. Therefore, we assessed differences in age, BMI, nationality, income, and plasma concentrations of selenium, copper, and zinc across prisons. Kruskal–Wallis tests were used for continuous variables with non-normal distributions, followed by Dunn’s *post hoc* tests with Bonferroni adjustment for multiple comparisons. Categorical variables were compared using chi-squared tests, followed by pairwise comparisons with Bonferroni correction.

#### Associations within the incarcerated population

Associations between selenium, copper, and zinc plasma levels and sociodemographic or health-related factors within the incarcerated population were assessed using univariable and multivariable linear regression models. Multivariable models adjusted for age, education level, pre-incarceration income, prison site, BMI, self-rated health, and smoking status. Self-rated health, collected on a visual analogue scale (0–100), was dichotomized at the median for analytical simplicity and comparability. Regression coefficients (β) with standard errors (SE) and p-values were reported, with two-sided statistical significance set at p < 0.05.

## Results

The study included 206 men with a mean age of 37.8 years (SD = 12.8). Among them, 61.0% (114/206) reported a pre-incarceration household income below 3,000 CHF, and 51.2% (104/203) had no education beyond compulsory schooling. A total of 72.1% (142/197) were of foreign origin, representing 50 different nationalities. These figures are consistent with national prison statistics from the FSO, particularly regarding age and the proportion of foreign nationals. Additional details are presented in Supplementary Table S1.

Plasma concentrations of selenium, copper and zinc among participants had median values of 106 ng/mL for Se, 911 ng/mL for Cu and 824 ng/mL for Zn (see Table 1). Substantial interindividual variability was observed, particularly for zinc and copper, with wide dispersion between the 5th and 95th percentiles (see Supplementary Table S2 for full distribution).

**TABLE 1:** Comparison of plasma selenium, copper and zinc concentrations between men in detention and men from the general population, with summary statistics and unadjusted and adjusted group comparisons (adjusted for age and/or body mass index). (Sérocovid Prison and NutriSerocovid studies, Vaud, Switzerland, 2023).

Characteristics	Men in detention (N = 196)^c^	Men from the general population (N = 189)^c^	Differences (CI95%)	P-value^b^	Wilcoxon rank-sum test (p-value^b^)
Age [mean (SD)]	37.8 (12.8)	57.3 (18.0)			
BMI [mean (SD)]	26.5 (5.73)	26.1 (3.75)			
Selenium					
< Lower reference limit^b^ [%, N]	11.7%, 23	8.47%, 16		0.37*	
Median [ng/mL], (IQR)	106 (23.9)	105 (18.2)		0.47**^#^	0.69
Age adjusted			−1.96 (−5.44, 0.44)	0.35***	
BMI adjusted			1.59 (−2.18, 4.02)	0.41***	
Age and BMI adjusted			−2.21 (−5.71, −0.11)	0.27***	
Mean [ng/mL], (SD)	105 (18.3)	106 (17.6)	−0.54 (−3.05, 4.14)	0.77****	
Age adjusted (CI 95%)	103 (101, 106)	107 (104, 110)	−3.85 (−8.07, 0.36)	0.07*****	
BMI adjusted (CI 95%)	105 (102, 107)	106 (103, 108)	−0.79 (−4.45, 2.86)	0.67*****	
Age and BMI adjusted (CI 95%)	103 (100, 106)	108 (105, 110)	−4.66 (−8.97, −0.35)	0.03*****	
Copper					
< Lower reference limit^b^ [%, N]	5.10%, 10	1.59%, 3		0.10*	
Median [ng/mL], (IQR)	911 (220)	905 (192)		0.76**^##^	0.65
Age adjusted			51.4 (28.5, 112)	0.07***	
BMI adjusted			3.19 (−27.1, 35.7)	0.88***	
Age and BMI adjusted			46.2 (27.4, 105)	0.08***	
Mean [ng/mL], (SD)	916 (207)	907 (153)	−8.42 (−44.7, 27.9)	0.65****	
Age adjusted (CI 95%)	939 (912, 966)	882 (854, 910)	57.0 (14.7, 99.3)	0.008*****	
BMI adjusted (CI 95%)	912 (886, 938)	908 (882, 934)	4.31 (−32.4, 41.0)	0.82*****	
Age and BMI adjusted (CI 95%)	935 (907, 963)	883 (855, 911)	51.8 (8.78, 94.8)	0.02*****	
Zinc					
< Lower reference limit^b^ [%, N]	6.12%, 12	9.52%, 18		0.29*	
Median [ng/mL], (IQR)	824 (217)	749 (131)		< 0.0001**^§^	**< 0.0001**
Age adjusted			32.6 (5.09, 75.2)	0.10***	
BMI adjusted			66.6 (42.9, 98.7)	0.0002***	
Age and BMI adjusted			27.5 (−4.37, 56.7)	0.18***	
Mean [ng/mL], (SD)	830 (157)	750 (109)	80.1 (−107, −53.1)	< 0.0001****	
Age adjusted (CI 95%)	811 (790, 831)	771 (751, 792)	39.3 (8.10, 70.6)	0.01*****	
BMI adjusted (CI 95%)	828 (809, 847)	750 (731, 770)	77.7 (50.1, 105)	< 0.0001*****	
Age and BMI adjusted (CI 95%)	807 (786, 827)	774 (753, 794)	33.1 (1.31, 65.0)	0.04*****	

^a^ The micronutrient reference ranges were: Zn 620–999 ng/mL, Se 86–144 ng/mL, Cu 594–1141 ng/mL. These ranges correspond to percentiles 5 and 95 from the SKIPOGH, study, a population-based cohort of adults recruited in Switzerland between 2009 and 2012. We excluded women and individuals under 18 years of age from the SKIPOGH, sample.

^b^ A p-value less than 0.05 signifies a statistically significant difference at the 5% level. Specifically:

* Proportions of Lower reference limit: p-values from chi-squared tests.

** Median differences: p-value from median test.

*** Median differences adjusted for age and/or BMI: p-values from quantile regression.

**** Unadjusted mean differences: p-values from *t*-tests comparing group means.

***** Adjusted mean differences: p-values from *t*-tests on regression model coefficients, indicating the group effect’s significance after adjusting for covariates.

^c^ N denotes sample size.

Wilcoxon rank-sum tests: ^#^ 0.69, ^##^ 0.65, § <0.0001.

Bold values refer to P values < 0.05.

**FIGURE 1:**
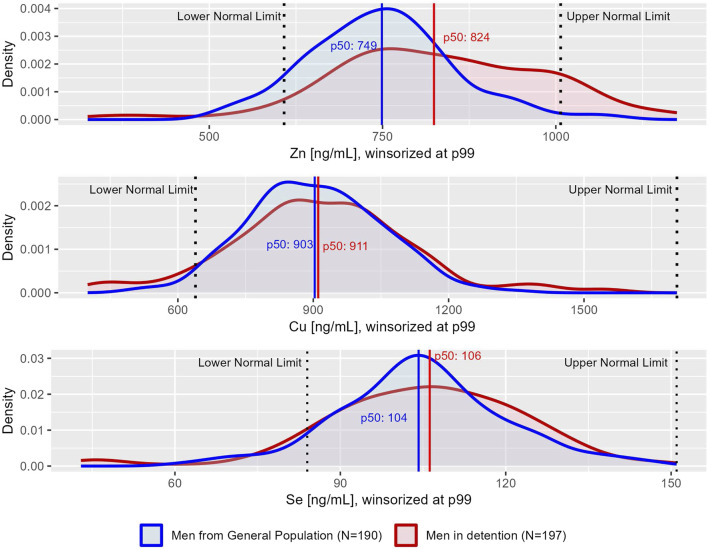
Distribution of plasma zinc, copper and selenium in men from the general population and men in detention (canton of Vaud, Switzerland). The upper panel shows the distribution (density) of plasma zinc levels (ng/mL) in men in detention (red line and area under the curve) and men in the general population (blue line and area under the curve). Similar distributions are presented in the middle panel for plasma copper (ng/mL) and in the lower panel for plasma selenium (ng/mL). In each of the three panels, the vertical blue line represents the median value for men in the general population, the vertical red line represents the median value for men in detention, and the vertical dotted lines represent the lower (left) and upper (right) normal limits. Data have been winzorized at percentile 99 (p99), meaning that values higher then percentile 99 have been replaced by the percentile 99 value, for each micronutriment. (Sérocovid Prison and NutriSerocovid studies, Vaud, Switzerland, 2023).

To place these values in context, we compared them to data from the NutriSerocovid study. As shown in Figure 1, the zinc distribution among incarcerated men appears shifted to the right and more dispersed compared to the general population. For copper and selenium, distributions were more similar, with overlapping medians and less pronounced differences in shape.

CRP levels, measured for all participants, were low and normal, except for one individual with a value of 20 mg/L: the 75th percentile was 2.33 mg/L. Given this limited variability, no adjustment for CRP was performed in the analysis.

Comparison of plasma concentrations between incarcerated men and community men revealed nuanced differences depending on the element and the adjustment factors applied (see Table 1). For selenium, median levels were nearly identical between groups (106 ng/mL in prison vs. 105 ng/mL in the general population), and neither unadjusted nor adjusted comparisons of medians showed significant differences. However, after adjusting for age and BMI, mean selenium levels were significantly lower among incarcerated men (adjusted mean difference: −4.66 ng/mL; 95% CI: −8.97 to −0.35; p = 0.03), a contrast not apparent in the unadjusted comparison (p = 0.77), suggesting a confounding effect of age.

For copper, both unadjusted and adjusted median and mean levels were slightly higher in the incarcerated population (median: 911 vs. 905 ng/mL; mean: 916 vs. 907 ng/mL), but the differences were not statistically significant until adjusting for age and BMI (see Table 1). After adjustment, mean copper levels were significantly higher among incarcerated individuals (adjusted mean difference: +51.8 ng/mL; 95% CI: 8.78 to 94.8; p = 0.02), again underscoring the influence of age on group comparisons.

For zinc, the difference was more pronounced. Incarcerated men had significantly higher median (824 vs. 749 ng/mL, p < 0.0001) and mean levels (830 vs. 750 ng/mL, p < 0.0001) compared to the general population (see Table 1). However, this difference was attenuated after adjustment for age and BMI, and only the adjusted mean remained statistically significant (adjusted mean difference: +33.1 ng/mL; 95% CI: 1.31 to 65.0; p = 0.04), while the adjusted median difference lost significance (p = 0.18).

These discrepancies between mean and median findings are visually supported by Figure 1, which shows a broader and right-skewed distribution of zinc values in the incarcerated population. Such distributional asymmetries likely contribute to the divergence in statistical significance between parametric and non-parametric tests. For copper and selenium, distributions appear more symmetric and overlapping, consistent with the less pronounced group differences observed.

The prevalence of concentrations below the Swiss lower reference limits did not differ significantly between groups for any element.

Using alternative cut-offs (700 ng/mL), the proportion of men with low plasma zinc was significantly higher in the community (17.3% vs. 32.3%; p < 0.001). For copper (636 ng/mL), low plasma copper was more frequent among detained men (7.65% vs. 1.59%; p = 0.01). For selenium (60 ng/mL), the proportion below the cut-off was low in both groups (2.55% vs. 0%; p = 0.08). Details are presented in Supplementary Table S3.

We also examined differences in sociodemographic characteristics and micronutrient levels across the three detention facilities (Table 2). Men in Prison 3 were significantly older and had a higher BMI compared to those in Prison 1 (p < 0.001 and p = 0.040, respectively), while no significant differences were observed between Prisons 1 and 2. The proportion of foreign-origin individuals was highest in Prison 2, which differed significantly from both other sites (p = 0.019 vs. Prison 1; p = 0.007 vs. Prison 3).

**TABLE 2:** Sociodemographic characteristics and plasma concentrations of zinc, copper and selenium among incarcerated men by prison facility, with overall tests and *post hoc* pairwise comparisons. (Sérocovid Prison Study, Vaud, Switzerland, 2023).

Characteristics	Prison 1 (N = 65)	Prison 2 (N = 61)	Prison 3 (N = 78)	p-value	Test post-hoc
Age (years), mean (SD)	33.4 (10.4)	37.7 (13.1)	41.7 (13.4)	**< 0.001** ^*^	P1 vs. P3 (p < 0.001)
BMI (kg/m^2^), mean (SD)	25.3 (4.47)	26.2 (4.67)	27.7 (7.06)	0.046^*^	P1 vs. P3 (p = 0.040)
No school certificate/mandatory school certificate, N (%)	32 (49.2%)	28 (45.9%)	38 (48.7%)	0.861^**^	​
Total household income per month before incarceration <3,000 (CHF), N (%)	38 (58.4%)	33 (54.1%)	43 (55%)	0.075^**^	​
Current smoker, N (%)	52 (80.0%)	46 (75.4%)	57 (73.1%)	0.4^**^	​
Foreign-origin, N (%)	39 (60.0%)	54 (88.5%)	49 (62.8%)	**0.003** ^**^	P2 vs. P1 (p = 0.019 P2 vs. P3 (p = 0.007)
Se (ng/mL), mean (SD)	100 (17.6)	108 (18.3)	106 (18.2)	0.07^*^	​
Cu (ng/mL), mean (SD)	884 (209)	887 (175)	965 (222)	**0.01** ^*^	P3 vs. P1 (p = 0.045) P3 vs. P2 (p = 0.021)
Zn (ng/mL), mean (SD)	838 (165)	783 (132)	857 (158)	**0.004** ^*^	P2 vs. P1 (p = 0.038) P2 vs. P3(p = 0.004)

* p-values from kruskal-Wallis, followed by Dunn’s post-hoc test with Bonferroni correction.

** p-values from chi-squared tests, followed by pairwise chi-squared tests with Bonferroni correction.

Bold values refer to P values < 0.05.

Micronutrient concentrations also varied across facilities (Table 2). Zinc levels were significantly lower in Prison 2 compared to both Prisons 1 and 3 (p = 0.038 and p = 0.004, respectively). Conversely, copper levels were significantly higher in Prison 3 than in Prisons 1 and 2 (p = 0.045 and p = 0.021). No significant differences were found for selenium.

Finally, to identify factors associated with micronutrient status, we conducted univariable and multivariable regression analyses. Tables 3–5 summarize associations between selenium, copper, and zinc levels and variables such as age, education, income, BMI, self-rated health, smoking, and prison site.

**TABLE 3:** Associations between plasma selenium concentrations and sociodemographic, behavioral and health-related factors among incarcerated men, based on bivariable and multivariable linear regression models (Sérocovid Prison Study, Vaud, Switzerland, 2023).

Selenium^d^	Bivariable^a^		Multivariable^a^	
​	β (SE)^e^	P-value^c^	β (SE)^e^	P-value^c^
Age (years)^d^	−0.11 (0.10)	0.28	−0.29 (0.13)	**0.02**
Education				
No school certificate/mandatory school certificate	1	​	1	1
Post-mandatory school certificate	2.62 (2.65)	0.32	5.86 (3.00)	**0.05**
Total household income per month before incarceration (CHF)				
≥3′000^b^	1	​	1	1
<3′000	−4.46 (2.78)	0.11	−7.65 (3.16)	**0.02**
Prison site				
Prison 1^b^	1	​	1	1
Prison 2	7.30 (3.27)	**0.03**	4.39 (3.79)	0.25
Prison 3	5.95 (3.10)	0.06	3.92 (3.68)	0.29
BMI (kg/m^2^)^d^	0.16 (0.23)	0.50	0.24 (0.28)	0.40
Self-rated health				
Above mediane^b^	1	​	1	​
Below mediane	−4.95 (2.69)	0.07	−2.79 (2.89)	0.34
Smoking status				
Non-smoker^b^	1	​	1	​
Current smoker	−5.04 (3.09)	0.105	−11.1 (3.53)	**0.02**

^a^ The table reports regression coefficients (**β**), standard errors (SE) and p-values from bivariable models and from a multivariable model including all listed covariates.

^b^ Reference categories are indicated by the value 1.

^c^ Statistical significance was defined as p < 0.05; p-values are reported consistently (e.g., p < 0.001).

^d^ Units: plasma selenium in ng/mL; age in years; body mass index in kg/m^2^.

^e^ Post-estimation: coefficients represent the absolute change in selenium (ng/mL) per one-unit increase in the covariate (continuous variables) or the mean difference versus the reference category (categorical variables).

Bold values refer to P values < 0.05.

**TABLE 4:** Associations between plasma copper concentrations and sociodemographic, behavioral and health-related factors among incarcerated men, based on bivariable and multivariable linear regression models (Sérocovid Prison Study, Vaud, Switzerland, 2023).

Copper^d^	Bivariable^a^		Multivariable^a^	
​	β (SE)^e^	P value^c^	β (SE)^e^	P value^c^
Age (years)^d^	1.99 (1.14)	0.08	2.04 (1.44)	0.16
Education				
No school certificate/mandatory school certificate	1	​	1	1
Post-mandatory school certificate	14.7 (29.7)	0.62	49.6 (33.4)	0.14
Total household income per month before incarceration (CHF)				
≥3′000^b^	1	​	1	1
<3′000	−45.6 (30.8)	0.14	−40.2 (35.4)	0.26
Prison site				
Prison 1^b^	1	​	1	1
Prison 2	3.49 (37.1)	0.92	−45.4 (42.2)	0.28
Prison 3	81.07 (35.2)	**0.02**	51.1 (41.0)	0.21
BMI (kg/m^2^)^d^	3.78 (2.58)	0.14	2.73 (3.17)	0.39
Self-rated health				
Above mediane^b^	1	​	1	1
Below mediane	9.68 (30.4)	0.75	−1.37 (32.3)	0.97
Smoking status				
Non-smoker^b^	1	​	1	1
Current smoker	16.0 (35.8)	0.65	47.1 (39.3)	0.23

^a^ The table reports regression coefficients (**β**), standard errors (SE) and p-values from bivariable models and from a multivariable model including all listed covariates.

^b^ Reference categories are indicated by the value 1.

^c^ Statistical significance was defined as p < 0.05; p-values are reported consistently (e.g., p < 0.001).

^d^ Units: plasma copperin ng/mL; age in years; body mass index in kg/m^2^.

^e^ Post-estimation: coefficients represent the absolute change in copper (ng/mL) per one-unit increase in the covariate (continuous variables) or the mean difference versus the reference category (categorical variables).

Bold values refer to P values < 0.05.

**TABLE 5:** Associations between plasma zinc concentrations and sociodemographic, behavioral and health-related factors among incarcerated men, based on bivariable and multivariable linear regression models (Sérocovid Prison Study, Vaud, Switzerland, 2023).

Zinc^d^	Univariable^a^		Multivariable^a^	
​	β (SE)^e^	P value^c^	β (SE)^e^	P value^c^
Age (years)^d^	−2.80 (0.85)	**0.001**	−3.30 (1.03)	**0.002**
Education				
No school certificate/mandatory school certificate	1	​	1	​
Post-mandatory school certificate	30.7 (22.6)	0.18	17.4 (24.1)	0.47
Total household income per month before incarceration (CHF)				
≥3′000^b^	1	​	1	​
<3′000	3.79 (23.4)	0.87	−39.2 (25.5)	0.13
Prison site				
Prison 1^b^	1	​	1	​
Prison 2	−55.4 (27.7)	**0.05**	−66.9 (30.1)	**0.03**
Prison 3	19.1 (26.3)	0.47	20.3 (29.5)	0.49
BMI (kg/m^2^)^d^	−0.03 (1.99)	0.99	1.77 (2.29)	0.44
Self-rated health				
Above mediane^b^	1	​	1	​
Below mediane	−19.4 (23.1)	0.41	−20.7 (23.8)	0.38
Smoking status				
Non-smoker^b^	1	​	1	​
Current smoker	27.6 (26.6)	0.30	−29.3 (28.3)	0.30

^a^ The table reports regression coefficients (β), standard errors (SE) and p-values from bivariable models and from a multivariable model including all listed covariates.

^b^ Reference categories are indicated by the value 1.

^c^ Statistical significance was defined as p < 0.05; p-values are reported consistently (e.g., p < 0.001).

^d^ Units: plasma zinc in ng/mL; age in years; body mass index in kg/m^2^.

^e^ Post-estimation: coefficients represent the absolute change in zinc (ng/mL) per one-unit increase in the covariate (continuous variables) or the mean difference versus the reference category (categorical variables).

Bold values refer to P values < 0.05.

For selenium, lower levels were associated with older age (β = −0.29, p = 0.02), low income (β = −7.65, p = 0.02), and current smoking (β = −11.1, p = 0.02). A borderline association was also observed for education (β = 5.86, p = 0.05). Selenium levels initially appeared higher in Prison 2, but this difference was not significant after adjustment for confounders.

For copper, no strong associations were identified. Although men in Prison 3 had higher copper levels in unadjusted models (β = 81.1, p = 0.02), this association did not remain significant after adjustment.

Zinc concentrations were significantly associated with age and prison site. Older men had lower zinc levels (β = −3.30, p = 0.002), and individuals in Prison 2 had significantly lower levels than those in Prison 1 (β = −66.9, p = 0.03). No other variables showed significant associations.

## Discussion

This study provides the first descriptive assessment of plasma zinc, copper, and selenium concentrations among incarcerated men in Switzerland. Overall, few men had plasma concentrations below reference limits and proportions did not differ significantly from those observed in men from the general population.

After adjustment for age and BMI, subtle differences emerged. Selenium levels were slightly lower, while zinc and copper were slightly higher among incarcerated men. These shifts were not significant in median-based comparisons, indicating distributional differences rather than consistent population-wide disparities. Zinc showed a more dispersed, right-skewed distribution, with greater interindividual variability, a pattern, confirmed by kernel density plots and Wilcoxon rank-sum test, suggesting heterogeneity within the study population.

Sensitivity analyses using alternative lower cut-offs made some between-group differences statistically significant, consistent with the distributional patterns described above. Selenium values below the cut-off were rare in both groups, copper was more often below the cut-off among detained men, and zinc below the cut-off increased in both groups but remained more prevalent among community men. These unadjusted findings should be interpreted cautiously, as they illustrate how the prevalence of values below selected thresholds depends on the cut-offs applied and highlight the need for clearer, outcome-oriented reference ranges.

Plasma selenium is considered as a suitable biomarker for ranking subjects according to long-term selenium intake [42]. Its informative value as a biomarker depends on overall selenium status but additional, yet costly, biomarkers such as selenoprotein would be needed for a comprehensive understanding of selenium status [43]. Lower selenium in the prison sample was associated with older age and smoking, consistent with community-based studies [44–49]. Lower socio-economic position, measured via pre-incarceration income, was also linked to lower selenium concentrations. However, we found no prior studies directly relating plasma selenium to income. Socioeconomic differences in diet quality are well documented and may contribute to differences in selenium intake. [50–53]. Slightly lower mean selenium concentrations compared with the general population may plausibly reflect the much higher smoking prevalence in prisons (76.8% current smoker in our sample), given its known negative association with selenium status [44–47, 49]. Smoking has also been linked to broader alterations in micronutrient status [49, 54–57].

In addition, dietary patterns and environmental factors, including the selenium content of food sources, influence selenium exposure. These factors may contribute to variability in selenium concentrations across populations. The absence of dietary intake data, detailed health indicators, and institutional dietary assessments limit the interpretation of observed micronutrient concentrations. In addition, plasma concentrations of trace elements, particularly zinc and copper, are influenced by physiological and inflammatory processes and therefore may not accurately reflect individual nutritional status; however, inflammation was not present in this case (median CRP = 2.3 mg/L).

There is currently no specific, reliable biomarker that indexes habitual zinc intake or total body zinc; marginal deficiency at the individual level is difficult to detect [35, 58]. Plasma zinc, the most used indicator, reflects only a small mobilizable pool and is highly sensitive to non-nutritional factors (infection, stress, inflammation) as well as fasting status and sampling time, with post-prandial decreases of up to ∼19% [31, 59–61]. Because fasting status and draw time were not recorded (CRP was generally low), some measurement variability cannot be excluded.

In our sample, zinc declined with age and was significantly lower in Prison 2. This facility primarily houses men in pretrial detention (a smaller proportion serving sentences), a stricter regime, and has been characterized by chronic overcrowding and suboptimal infrastructure, conditions likely to heighten stress. Shorter stays also mean the effects of pre-incarceration vulnerabilities (e.g., poor diet, substance use) may still be present. These factors should be interpreted cautiously, as similar constraints affect individuals detained in Prison 1. Dietary differences could also contribute: Prisons 1 and 3 share a centrally prepared menu, unlike Prison 2, which operates its own. Together, these contextual features may help explain lower zinc measured in Prison 2. It is possible that unmeasured factors, such as incarceration duration, stress and canteen use, explain part of the observed differences between prisons.

Plasma copper was not associated with available covariates in our analyses. Slightly higher mean levels among incarcerated men, but still within normal ranges, can be considered as non-clinically relevant and may reflect behavioral or health factors. Smoking, strongly prevalent in prisons, has been linked to higher plasma copper through increased ceruloplasmin synthesis [49, 57], although in our data smoking was not a statistically significant predictor. Higher values are associated with cardiovascular disease [62], but this study is not adequately powered to explore such link. Other unmeasured factors such as inflammation (which was not present in the study) [37] or liver disease [63–65] also contribute, and the clinical relevance of this finding remains uncertain.

### Strengths and limitations

This study provides novel biomarker-based data on trace element concentrations in an understudied population, for which such data remain scarce. Strengths include a relatively large sample size and the inclusion of participants from three prison facilities within the canton of Vaud, enhancing the diversity of the study population. Furthermore, comparison with data from the general population provides important contextual insight into the observed distribution.

Limitations include cross-sectional design which precludes causal inference as well as disentangling between pre-incarceration factors and prison-related factors. In addition, no information was available on duration of incarceration, precluding causal inference; the restricted adjustment (age and BMI only) limited with comparison with the general population studies in NutriSerocovid [38]: the adjusted differences should be interpreted with caution, especially for selenium and copper. We also lacked data on fasting status, with blood draws made during the morning before 12AM, with no precise relation to food intake, a limitation particularly relevant for zinc [60]. Finally, the study included only men, limiting generalizability to women. However, as men constitute 94.1% of the incarcerated population, findings remain broadly representative of prison population. International evidence on biomarker-based micronutrient status in prisons is sparse. Data exist for vitamin D [66] and, to a lesser extent, iron, with isolated reports on selenium [67]. We identified one recent Canadian prison study measuring serum zinc and copper, but it focused on impulsivity/ADHD and reported only between-group differences [68]. Most prison nutrition literature instead assesses diet quality or menu nutrient content [12, 69, 70]. This scarcity is both a limitation, constraining external comparability, and a strength, as our study addresses this gap by providing a multi-micronutrient biomarker profile in a Western European prison context.

Additional limitations affect interpretation of the findings. Pre-analytical factors, including fasting status and timing of blood collection, were not recorded. Therefore, part of the observed variability in plasma concentrations may reflect methodological rather than biological differences. This may be particularly important for plasma zinc levels, which are known to be influenced by fasting condition, inflammatory status and time of the day [60]. Note however that zinc levels are higher during fasting conditions than after a meal [60]. Failure to account for fasting status may lead to an overestimation of zinc deficiency. Considering that the proportion of zinc levels below the population threshold in prison were low and like what we observed in men from the general population, this further reinforces the message that there is no worrying zinc deficiency in the prison setting in the canton of Vaud.

The above-mentioned absence of dietary intake data, detailed health indicators, and institutional dietary assessments restrict the interpretation of observed micronutrient concentrations. Finally, the study included only men, limiting generalizability to women; however, men represent the majority of the incarcerated population.

### Public health implications and conclusion

Our findings are cautiously reassuring. Incarcerated men in the canton of Vaud showed no excess rates of zinc, copper, or selenium below reference limit, and overall concentrations were within population-based ranges. Broader zinc distribution, lower levels in one pretrial facility, and the slightly reduced selenium suggest that both individual vulnerabilities and institutional conditions may influence micronutrient status.

These results are particularly relevant in correctional settings, where the burden of infectious diseases such as HIV, HCV, tuberculosis, and more recently COVID-19 is elevated. Selenium, zinc, and copper all play important roles in immune function, and even modest differences may affect resilience against infection.

In Switzerland, prison food is subject to external oversight by the National Commission for the Prevention of Torture and the European Committee for the Prevention of Torture. Their visits provide qualitative assessments and have not flagged systematic deficiencies [26, 71]. However, these reports are not formal nutritional audits and do not include detailed menu analyses against dietary standards. Moreover, official assessments do not capture actual consumption, which is shaped by access to canteen products and by the social and symbolic functions of food in prison life. Protests, such as the 2014 meal boycott at a prison in Vaud, illustrate that food embodies more than nutrition, complicating oversight and interventions [72–74].

Safeguarding nutritional health in prisons is both a matter of equity and public health issue, as individuals in detention eventually return to the community. Continued monitoring is warranted to identify at-risk groups, account for institutional differences, and ensure that prison food systems support both health and wellbeing [75, 76].
