# Supplementary material for: Plasma levels of zinc, selenium, and copper among incarcerated men in Switzerland
Source: Int J Public Health. 2026 Aug 4;71:1609553. doi: 10.3389/ijph.2026.1609553 (PMC13482155; doi:10.3389/ijph.2026.1609553)
Supplement: Supplementary file 1 [file Supplementaryfile1.docx]

## **Supplementary materials**

**Table S1**. Characteristics of study participants (Sérocovid Prison Study, Vaud, Switzerland, 2023)

| **Variables** | **N** | **%** | **Missing^a^** |
| --- | --- | --- | --- |
| Age (years) |  |  | 0 |
| 18-39 | 121 | 59.0 |  |
| 40-64 | 76 | 37.1 |  |
| ≥ 65 | 8 | 3.9 |  |
| Highest education completed |  |  | 2 |
| No school certificate or mandatory school certificate | 104 | 51.2 |  |
| Post-mandatory school certificate | 99 | 48.8 |  |
| Total household income per month before incarceration (CHF) |  |  | 18 |
| < 3'000 | 114 | 61.0 |  |
| ≥ 3’000 | 73 | 39.0 |  |
| Prison site |  |  | 1 |
| Prison 1 | 78 | 38.2 |  |
| Prison 2 | 65 | 31.9 |  |
| Prison 3 | 61 | 29.9 |  |
| Weight Status^b^ |  |  |  |
| Underweight | 3 | 1.5 |  |
| Normal Range | 95 | 48.0 |  |
| Overweight | 61 | 30.8 |  |
| Obese | 39 | 19.7 |  |
| Smoking status |  |  | 2 |
| Current smoker | 156 | 76.8 |  |
| Non-smoker | 47 | 23.2 |  |
| Self-rated health^c^ |  |  | 6 |
| (Very) Good | 26 | 13.1 |  |
| Average | 54 | 27.1 |  |
| (Very) Bad | 119 | 59.8 |  |
| Nationality |  |  | 8 |
| Foreign-origin | 142 | 72.1 |  |
| Swiss-origin | 55 | 27.9 |  |

^a^The Missing column indicates non-responses for each variable

^b^Weight status is based on body mass index (BMI): underweight <18.5 kg/m²; normal range 18.5-24.9 kg/m²; overweight 25.0-29.9 kg/m²; obese ≥30.0 kg/m²

^c^Self-rated health was assessed on a scale from 0 to 100%, with 100 representing the best possible health status. Categories were defined as follows: (Very) Good (100-61), Average (60-41), and (Very) Bad (40-0).

**Table S2.** Distribution of plasma zinc, copper and selenium among incarcerated men, reported as minimum, maximum and the 5th, 25th, 50th, 75th and 95th percentiles*.* (Sérocovid Prison Study, Vaud, Switzerland, 2023)

| Variable | Min | p5 | p25 | p50 | p75 | p95 | Max | N^a^ |
| --- | --- | --- | --- | --- | --- | --- | --- | --- |
| Se (ng/ml) | 43.0 | 77.3 | 93.7 | 106 | 117 | 133 | 150 | 196 |
| Cu (ng/ml) | 400 | 593 | 801 | 911 | 1021 | 1197 | 1871 | 196 |
| Zn (ng/ml) | 325 | 602 | 730 | 824 | 946 | 1052 | 1326 | 196 |

^a^N denotes sample size

**Table S3.** Proportion of men with plasma selenium, copper, and zinc below the lower reference limit (LRL), comparing men in detention with men from the general population, using different tresholds: population-based limits from the Swiss Kidney project on genes in hypertension (SKIPOGH) and alternative cut-offs (Sérocovid Prison and NutriSerocovid Studies, Vaud, Switzerland, 2023)

| Micronutriment | Men in detention (N=196) | Men from the general population (N=189) | P-value* |
| --- | --- | --- | --- |
| Selenium |  |  |  |
| < LRL [%, N] SKIPOGH | 11.7%, 23 | 8.47%, 16 | 0.37* |
| < LRL [%, N] ESPEN/STEMDR | 2.55%, 5 | 0%, 0 | 0.08 |
| Copper |  |  |  |
| < LRL [%, N] SKIPOGH | 5.10%, 10 | 1.59%, 3 | 0.10* |
| < LRL [%, N] STEMDR | 7.65%, 15 | 1.59% 3 | **0.01** |
| Zinc |  |  |  |
| < LRL [%, N] SKIPOGH | 6.12%, 12 | 9.52%, 18 | 0.29* |
| < LRL [%, N] IziNCG/BOND | 17.3%, 34 | 32.3%, 61 | **< 0.001** |

*^a^* SKIPOGH lower reference level: Zn 620 ng/mL, Se 86 ng/mL, Cu 594 ng/mL. These cut-offs correspond to the 5th and 95th percentiles from the SKIPOGH study, a population-based cohort of adults recruited in Switzerland between 2009 and 2012. Women and individuals under 18 years of age were excluded from the SKIPOGH sample.

*^b^* Alternative cut-offs: Se 60ng/ml (ESPEN clinical threshold; GPx plateau; also used by STEMDR); Cu 636 ng/mL (STEMDR); Zn 700 ng/mL (IZiNCG/BOND; close to STEMDR 718 ng/mL).

*^c^* P-values for proportions < LRL of are based on chi-squared tests comparing men in detention with men from the general population.

*^d^* N denotes sample size.

* A p-value less than 0.05 was considered statistically significant at the 5% level.
